# ‘High desire’, or ‘merely’ an addiction? A response to Steele et al.

**DOI:** 10.3402/snp.v4.23833

**Published:** 2014-02-21

**Authors:** Donald L. Hilton

**Affiliations:** Department of Neurosurgery, The University of Texas Health Sciences Center at San Antonio, USA

The validity of an argument depends on the soundness of its premises. In the recent paper by Steele et al., conclusions are based on the initial construction of definitions relating to ‘desire’ and ‘addiction’. These definitions are based on a series of assumptions and qualifications, the limitations of which are acknowledged by the authors initially, but inexplicably ignored in reaching the firm conclusions the authors make. Yet, the firmness of these conclusions is unwarranted, not only as a result of conceptually problematic initial premises but also due to problematic methodology.

Consider, for instance, the concept of ‘sexual desire’. The first paragraph acknowledges that ‘sexual desires must be consistently regulated to manage sexual behaviors’, and must be controlled when either illegal (pedophilia) or inappropriate (infidelity). The paragraph ends with the inference that the term ‘sexual addiction’ does not describe a problematic entity per se, but that it merely describes a subset of individuals with high levels of desire.

The next paragraph references a paper by Winters et al., which suggests that ‘dysregulated sexuality … may simply be a marker of high sexual desire and the distress associated with managing a high degree of sexual thoughts, feelings, and needs’ (Winters, Christoff, & Gorzalka, 2010). It is based on these assumptions that Steele et al. then proceeds to question a disease model for this ‘distress’ associated with controlling sexual ‘desire’. For a comparison of different ‘desire’ templates, television viewing in children is used as an example. The last two sentences in this paragraph establish the premise that the rest of the paper then tries to prove:

Treatments focus on reducing the number of hours viewing television behaviorally without a disease overlay such as ‘television addiction’ and are effective. This suggests a similar approach might be appropriate for high sexual desire *if* the proposed disease model does not add explanatory power beyond merely high sexual desire. (Steele, Staley, Fong, & Prause, 2013)

Based on this comparison, that of desire to watch TV in children and desire for sex in adults, the authors then launch into a discussion on event-related potentials (ERPs) and a subsequent description of their study design, followed by results and discussion, and culminating in the following summary:

In conclusion, the first measures of neural reactivity to visual sexual and non-sexual stimuli in a sample reporting problems regulating their viewing of similar stimuli fail to provide support for models of pathological hypersexuality, as measured by questionnaires. Specifically, differences in the P300 window between sexual and neutral stimuli were predicted by sexual desire, but not by any (of three) measures of hypersexuality. (Steele et al., 2013)

With this statement the authors put forward the premise that high desire, even if it is problematic to those who experience it, is not pathologic, no matter the consequence.

Others have described significant limitations of this study. For instance, author Nicole Prause stated in an interview, ‘Studies of drug addictions, such as cocaine, have shown a consistent pattern of brain response to images of the drug of abuse, so we predicted that we should see the same pattern in people who report problems with sex if it was, in fact, an addiction’. John Johnson has pointed out several critical issues with this use of the Dunning et al. (2011) paper she cites as a basis for comparison with the Steele et al. paper. First, the Dunning et al. paper used three controls: abstinent cocaine users, current users, and drug naïve controls. The Steele et al. paper had no control group of any kind. Second, the Dunning et al. paper measured several different ERPs in the brain, including early posterior negativity (EPN), thought to reflect early selective attention, and late positive potential (LPP), thought to reflect further processing of motivationally significant material. Furthermore, the Dunning study distinguished the early and late components of the LPP, thought to reflect sustained processing. Moreover, the Dunning et al. paper distinguished between these different ERPs in abstinent, currently using, and healthy control groups. The Steele et al. paper, however, looked only at one ERP, the p300, which Dunning compared to the early window of the LLP. The Steele et al. authors even acknowledged this critical flaw in design: ‘Another possibility is that the p300 is not the best place to identify relationships with sexually motivating stimuli. The slightly later LPP appears more strongly linked to motivation’. Steel et al. admit that they are in fact not able to compare their results to the Dunning et al. study, yet their conclusions effectively make such a comparison. Regarding the Steele et al. study, Johnson summarized, ‘The single statistically significant finding says nothing about addiction. Furthermore, this significant finding is a *negative* correlation between P300 and desire for sex with a partner (r=−0.33), indicating that P300 amplitude is related to *lower* sexual desire; this directly contradicts the interpretation of P300 as *high* desire. There are no comparisons to other addict groups. There are no comparisons to control groups. The conclusions drawn by the researchers are a quantum leap from the data, which say nothing about whether people who report trouble regulating their viewing of sexual images have or do not have brain responses similar to cocaine or any other kinds of addicts’ (personal communication, John A. Johnson, PhD, 2013).

Although other serious deficiencies in this study design include lack of an adequate control group, heterogeneity of study sample, and a failure to understand the limitations of the ability of the P300 to qualitatively and quantitatively discriminate and differentiate between ‘merely high sexual desire’ and pathologically unwanted sexual compulsions, perhaps the most fundamental flaw relates to the use and understanding of the term ‘desire’. It is clear that in constructing this definitional platform, the authors minimize the concept of desire with the word ‘merely’. Desire, as related to biological systems in the context of sexuality, is a complex product of mesencephalic dopaminergic drive with telencephalic cognitive and affective mediation and expression. As a primal salience factor in sex, dopamine is increasingly recognized as a key component in sexual motivation, which has been widely conserved in the evolutionary tree (Pfaus, 2010). Genes relating to both the design and expression of sexual motivation are seen across phyla and also span intra-phyla complexity. While there are obvious differences between sex, food seeking, and other behaviors, which are essential to evolutionary fitness, we now know there are similarities in the molecular machinery from which biologically beneficial ‘desire’ emanates. We now know that these mechanisms are designed to ‘learn’, in a neural connecting and modulating way. As Hebb's law states, ‘Neurons that fire together, wire together’. We became aware of the brain's ability to alter its structural connectivity with reward learning in early studies relating to drug addiction, but have now seen neuronal reward-based learning with such seemingly diverse natural desires relating to sex and salt craving.

Definitions relating to desire are important here; biological salience, or ‘wanting’, is one thing, whereas we consider ‘craving’ to have more ominous implications as it is used in the literature relating to drug addiction and relapse. Evidence demonstrates that craving states relating to appetites for biologically essential necessities such as salt and sex invoke – with deprivation followed by satiation – a neuroplastic process involving a remodeling and arborizing of neuronal connections (Pitchers et al., 2010; Roitman et al., 2002). Notably, a desperate desire is effected by craving states associated with conditions that portend the possible death of the organism such as salt deficiency, which induces the animal to satiate and avoid death. Drug addiction in humans, interestingly, can affect a comparable craving leading to a similar desperation to satiate in spite of the risk of death, an inversion of this elemental drive. A similar phenomenon occurs with natural addictions as well, such as the individual with morbid obesity and severe cardiac disease continuing to consume a high fat diet, or one with a sexual addiction continuing to engage in random sexual acts with strangers despite an elevated probability of acquiring sexually transmitted diseases such as HIV and hepatitis. That gene sets driving signaling cascades essential to this craving conundrum are identical for both drug addiction and the most basic of natural cravings, salt, supports a hijacking, usurping role for addiction (Liedtke et al., 2011). We also better understand how complex systems associated with and effecting these changes involve genetic molecular switches, products, and modulators such as DeltaFosB, orexin, Cdk5, neural plasticity regulator activity-regulated cytoskeleton-associated protein (ARC), striatally enriched protein tyrosine phosphatase (STEP), and others. These entities form a complex signaling cascade, which is essential to neural learning.

What we experience affectively as ‘craving’, or very ‘high desire’, is a product of mesencephalic and hypothalamic impetus which projects to, participates in, and is part of cortical processing resulting from this convergence of conscious and unconscious information. As we demonstrated in our recent PNAS paper, these natural craving states ‘likely reflect usurping of evolutionary ancient systems with high survival value by the gratification of contemporary hedonic indulgences’ (Liedtke et al., 2011, PNAS), in that we found that these same salt ‘craving’ gene sets were previously associated with cocaine and opiate addiction. The cognitive expression of this ‘desire’, this focus on getting the reward, the ‘craving’ to experience satiation again is but a conscious ‘cortical’ expression of a deeply seated and phyolgenetically primitive drive originating in the hypothalamic/mesencephalic axis. When it results in an uncontrolled and – when expressed – destructive craving for a reward, how do we split neurobiological hairs and term it ‘merely’ high desire rather than addiction?

The other issue relates to immutability. Nowhere in the Steele et al. paper is there a discussion as to why these individuals have ‘high desire’. Were they born that way? What is the role, if any, of environment on both qualitative and quantitative aspect of said desire? Can learning affect desire in at least some of this rather heterogeneous study population? (Hoffman & Safron, 2012). The authors’ perspective in this regard lacks an understanding of the process of constant modulation at both cellular and macroscopic levels. We know, for instance, that these microstructural changes seen with neuronal learning are associated with macroscopic changes as well. Numerous studies confirm the importance of plasticity, as many have compellingly argued: ‘Contrary to assumptions that changes in brain networks are possible only during critical periods of development, modern neuroscience adopts the idea of a permanently plastic brain’ (Draganski & May, 2008); ‘Human brain imaging has identified structural changes in gray and white matter that occur with learning … learning sculpts brain structure’ (Zatorre, Field, & Johansen-Berg, 2012).

Finally, consider again the author's term ‘merely high sexual desire’. Georgiadis (2012) recently suggested a central dopaminergic role for humans in this midbrain to striatum pathway. Of all the natural rewards, sexual orgasm involves the highest dopamine spike in the striatum, with levels up to 200% of baseline (Fiorino & Phillips, 1997), which is comparable with morphine (Di Chiara & Imperato, 1988) in experimental models. To trivialize, minimize, and de-pathologize compulsive sexuality is to fail to understand the central biological role of sexuality in human motivation and evolution. It demonstrates a naiveté with regard to what is now an accepted understanding of current reward neuroscience, in that it pronounces sexual desire as inherent, immutable, and uniquely immune from the possibility of change either qualitatively or quantitatively. Even more critically, however, as illustrated by the Steele et al. paper, is that this myopic dogma fails to comprehend the truth that neuroscience now tells us that ‘high desire’, when it results in compulsive, unwanted, and destructive behavior, is ‘merely’ an addiction.
